# Regulation of the hypoxic tumor environment in hepatocellular carcinoma using RNA interference

**DOI:** 10.1186/s12935-016-0374-6

**Published:** 2017-01-03

**Authors:** Sung Hoon Choi, Jun Yong Park

**Affiliations:** 1 Division of Bioconvergence Analysis, Drug and Disease Target Group, Korea Basic Science Institute, Daejeon, Korea; 2 Department of Internal Medicine, Institute of Gastroenterology, Yonsei University College of Medicine, Seoul, Korea

**Keywords:** RNAi, Hepatocellular carcinoma, HIF, IL-8, Angiogenesis, Apoptosis

## Abstract

**Objectives:**

Hypoxia is the condition where tumor cells have been deprived of oxygen and has been shown to have a role of tumor development in the hepatocellular carcinoma (HCC).

**Methods:**

Using PubMed online database and Google scholar web site, the terms “angiogenesis”, “apoptosis”, “RNA interference” and/or “hepatocellular carcinoma (HCC)” were searched and analyzed.

**Results:**

The hypoxia inducible factors (HIFs) are transcriptional regulators that affect a homeostatic response to oxidative stress and have been identified as a key transcription activator of angiogenesis, survival, and metabolism. Cytokines, such as IL-8, also controlled endothelia cells survival and angiogenesis. IL-8 was also overexpressed under hypoxia and induced tumor angiogenesis and growth.

**Conclusion:**

Therefore, regulation of HIFs and IL-8 controlled the tumor microenvironment in terms of tumor angiogenesis and apoptosis. The review summarizes the results of regulation of the hypoxic tumor environment.

## Background

Hepatocellular carcinoma (HCC) is the sixth most common cancer in the world [1]. HCC is the second most lethal cancer [1]. About 80–90% of cirrhotic liver disease due to chronic viral hepatitis B (HBV) or C (HCV) develops into HCC [1]. Moreover, most cases of advanced HCC develop hypoxic induction with angiogenesis and growth programs [2].

The most important factors that impact HCC progression are oxygen and nutrients [3]. The liver is an organ with a specific blood supply. Approximately 25 and 75% of the blood enters the liver through the hepatic artery and the portal vein, respectively. The latter drains into smaller-diameter structures called sinusoids. Vascular resistance is very low in these structures, and portal venous blood, which is loaded with food and many microbial antigens from the intestine, flows extremely slowly into the sinusoids. Thus, large amounts of nutrients and oxygen are required for HCC cell proliferation, which results in localized hypoxia [3, 4]. This hypoxia environment causes tumor angiogenesis, which is the generation of new blood vessels from already existing ones [3, 5]. Tumor angiogenesis overcomes oxidative stress and deficiency of oxygen-dependent energy production caused by hypoxia [3]. The key factors responsible for the regulation of angiogenesis during hypoxia are HIF-1α and VEGF [5, 6]. However, various studies have reported that angiogenesis is induced even during inhibition of HIF-1α during hypoxia [7], and these findings demonstrate that tumor angiogenesis is also partially recovered by various other factors [7]. The increase in expression of various factors such as growth factor and tumor stimulating factors during hypoxia can induce angiogenesis, as they increase proliferation and ensure stabilization of endothelial cells, which are not only caused by HIF-1α, but also by cytokines such as interleukin IL-8, or other growth factors, such as PDGF [8, 9]. They also increase VEGF expression as well as contribute to the increase and stabilization of angiogenesis by stimulating of VEGFR on the surface of endothelial cells [10]. Moreover, various solid tumors pass through the following three stages during their reproduction cycle: cell proliferation, hypoxia, and recovery by angiogenesis [3].

### Role of HIF and IL-8 under hypoxia

The hypoxia inducible factors (HIFs) are a family of heterodimeric transcription factors that act as master regulators of a homeostatic transcriptional response to hypoxia in virtually all cells and tissues [3]. Active HIF consists of an alpha subunit and a beta subunit [2, 11, 12]. Three alpha subunits, termed HIF1a, HIF2a, and HIF3a, have been described in humans, mice, and rats; all bind to a common b subunit named, alternatively, HIF1b, or the aryl-hydrocarbon-nuclear receptor translocator (ARNT) [13, 14]. Active HIF is termed by its alpha subunit; hence, HIF1 is the active transcription factor consisting of HIF1α and ARNT, HIF2 is the dimer of HIF2α and ARNT, etc. [15]. HIF1 and HIF2 are the major hypoxia-inducible factors in humans, mice, and rats [13].

Under conditions of normoxia, HIF-1α subunits are hydroxylated at proline residues by hydroxylase enzymes [3, 16]. Hydroxylation of HIF1α and assembly on a protein scaffold consisting of the VHL tumor suppressor [17–20], along with other cofactors, result in the rapid ubiquitination of the alpha subunit and subsequent degradation by the proteasome. Conversely, in conditions of hypoxia, HIFα subunits escape degradation and are free to dimerize with the binding partner, ARNT [3, 13]. The HIF trans-locates to the nucleus and affects transcription of target genes, typically by binding to a hypoxia response element (HRE) in the upstream promoter region of the target genes such as, angiogenesis, apoptosis, metabolism, survival related genes etc. [2, 3, 14].

Interlukine-8 (CXCL-8, IL-8) is a key factor of endothelial cell survival and angiogenesis [21]. IL-8 is also regulated under hypoxia and directly controlled endothelial cell [22–25]. IL-8 has been shown to regulate pathological angiogenesis, tumor growth, and metastasis [24]. The mechanism(s) regulating IL-8-mediated endothelial cell survival are not well understood. Recent reports suggest that in addition to cell proliferation and migration, endothelial cell survival and death are also important components for tumor survival and development [25]. The other studies have shown that a cell cycle-regulated apoptosis inhibitor, survivin, and the cell death-related gene family products, Bcl-xl and Bcl-2 [15, 26], are associated with vascular endothelial growth factor (VEGF)-induced angiogenesis [10, 25]. IL-8 and its receptors CXCR1 and CXCR2 have been observed in endothelial cells and have been shown to play a role in endothelial cell proliferation [25]. Liver cancer, such as HCC, are dependent on angiogenesis; therefore, angiogenesis inhibition can be used as a potential treatment modality to inhibit the proliferation and growth of solid tumors [27, 28]. In addition, efforts to treat solid tumors using angiogenesis inhibitors have yielded good results [28, 29]. However, these therapies not only affect solid tumors but also normal cells, which is an area of concern in cancer treatment [27]. Furthermore, cancer therapies, such as transarterial chemoembolization (TACE) that uses blood vessels may not produce the desired results, and this may even increase vascular proliferation and growth into a malignant tumor by incomplete responses [30]. A correlation between hypoxia, cancer proliferation, and angiogenesis and the mechanism of growth or development of tumors has been observed [31]. If the link between any of the above can be elucidated, the basis for inhibition of tumor growth and excision can be ascertained. This can be achieved by dual control of HIF-1α and angiogenic factors [6]. Innovative and more effective cancer therapies can be developed by regulating HIF-1α expression, which is the key factor in hypoxia, and controlling the expression of IL-8 and other angiogenic stimulators, which restore the angiogenic processes, during inhibition of HIF-1α expression [7].

HIF-1α knockdown directly repressed tumor growth, whereas IL-8 knockdown indirectly repressed tumor growth [1, 7, 27]. Combined knockdown of HIF-1α and IL-8 increased survival rates of mice [7]. Conditioned media of Combined knockdown in HCC cells also decreased micro-vessel density and tumor volume in vivo [7]. Similarly, HIF-1α and IL-8 knockdown inhibited the angiogenic effects of HCC cell-conditioned media on tube formation and invasion by endothelial cells in vitro [7]. Inhibition of HIF-1α and IL-8 up-regulated the expression of apoptotic factors while down-regulating anti-apoptotic factors simultaneously [7]. Knockdown of HIF-1α and IL-8 increased concentration of cytosolic cytochrome C and enhanced DNA fragmentation in HCC cell lines and HUVECs [32]. Moreover, culture supernatant collected from the knockdown of HIF-1α and IL-8 in HCC cell lines induced apoptosis in HUVECs under hypoxia [32]. The silencing of HIF-1β expression suppressed tumor cell growth and inhibited the expression of tumor growth-related factors [33], such as vascular endothelial growth factor, epidermal growth factor, and hepatocyte growth factor. Suppression of tumor cell invasion and migration was also demonstrated in HIF-1β-silenced HCC cell lines [33].

### Inhibition of HIF-1α and IL-8 expression to suppress angiogenesis

Angiogenesis is essential for tumor growth and metastasis, and attempts to control tumor-associated angiogenesis may prove to be promising tactics for limiting progression [27]. Angiogenesis occurs during development and vascular remodeling as a controlled series of events leading to neovascularization, which supports changing tissue requirements [34]. Blood vessels and stromal components are responsive to pro- and anti-angiogenic factors that allow vascular remodeling during development, wound healing, and pregnancy [35, 36]. However, in pathological situations, such as cancer, the same angiogenic signaling pathways are induced and exploited.

Although an oncogenic event may allow tumor cells to evade surveillance or may enhance their survival, large-scale growth of a tumor ultimately requires a blood supply [27]. To obtain this blood supply, tumor cells can tilt the balance toward stimulatory angiogenic factors in order to drive vascular growth by attracting and activating cells from within the microenvironment of the tumor [37]. The magnitude and quality of the angiogenic response is ultimately determined by the sum of pro- and anti-angiogenic signals (Table 1) and, more specifically, their unique activities on multiple cell types [38]. Understanding how these various components are regulated is required for the design and development of effective anti-angiogenic therapies for cancer [27].

**Table 1:** Up regulation of tumor angiogenic factors under hypoxia

Factors	Functions	Ref
VEGF	Increased vascular permeability	[5]
	Endothelial sprouting	[36]
	EC proliferation and migration	[10, 35]
	EC assembly	[40]
iNO	Increased vascular permeability	[35]
Angiopoietin-2	Endothelial sprouting	[36]
PDGF	Pericyte recruting	[9, 56]
	EC proliferation and migration	[3, 40]
MMPs	Degradation of extracellular matrix	[5]
Tie2	Form the blood vessels	[40]
TGF-beta	Increased EC differentiation formation of vascular structure	[35, 40]
	Formed vascular structure	[10, 40]
VEGFR2	Stimulate endothelial cell mitogenesis and cell migration	[3, 10]
	Enhances microvascular permeability	[5, 56]
Endothelin	Regulates local vascular tone and integrity	[10, 40]
	Influences EC growth and survival	[5]
IL-8	Increased vascular permeability	[5]
	EC proliferation and migration	[25]
	Endothelial sprouting	[36]

In cancer, multiple sources and modes of vascular remodeling contribute to disease progression [28]. Targeting one aspect of this remodeling process may produce a short-term effect; nevertheless, suppressing one pathway could promote another [7]. The redundancy and diversity by which blood vessels can remodel might account for the poor efficacy or acquired resistance often observed in antiangiogenic therapies [3]. Improving therapeutic responses thus requires consideration of the signaling pathways that regulate the multiple cell types involved in the vascular components of cancer [39]. Once a tumor lesion exceeds a few millimeters in diameter, hypoxia and nutrient deprivation triggers an “angiogenic switch” to allow the tumor to progress [3, 15, 27]. Tumor cells exploit their microenvironment by releasing cytokines and growth factors to activate normal, quiescent cells around them, initiating a cascade of events that quickly becomes dys-regulated [40].

**Fig. 1:**
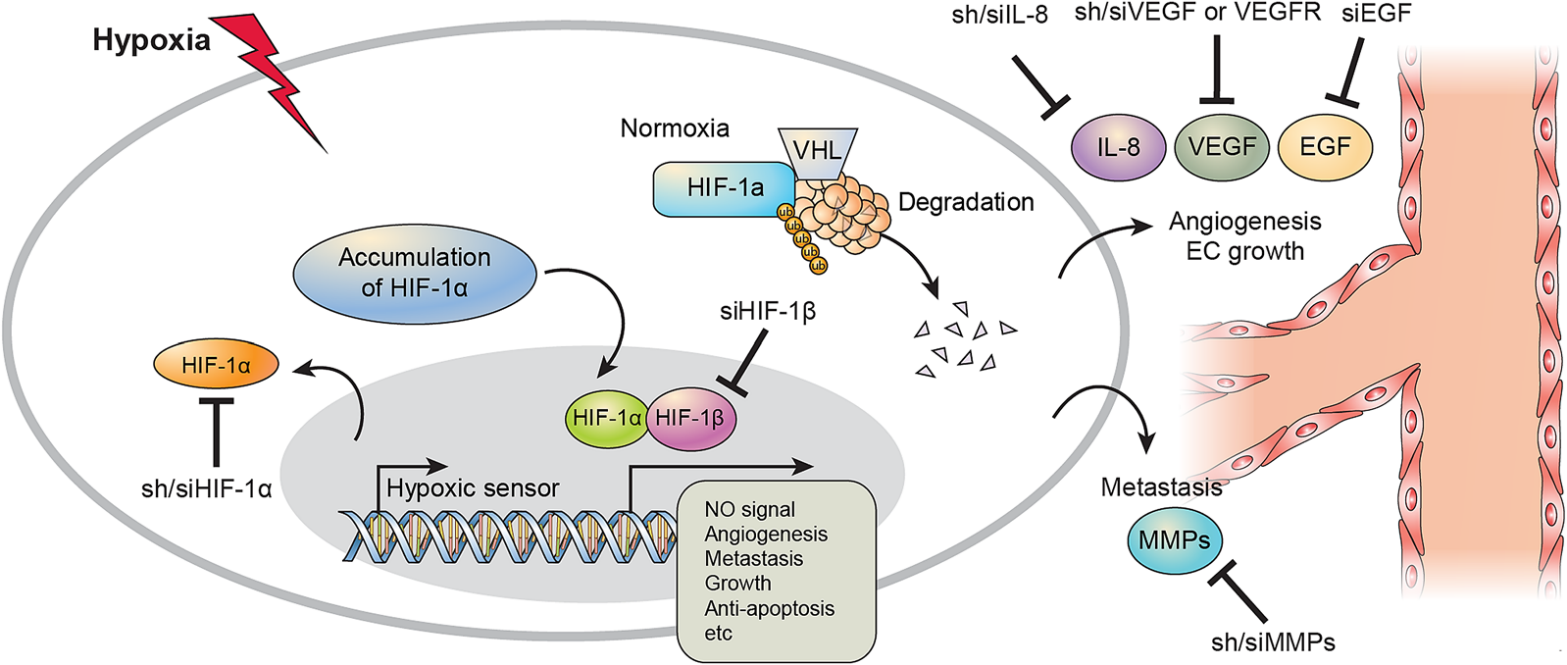
Inhibition of tumor angiogenesis by silencing of HIF-1α and IL-8. HIF-1α directly regulates HCC development and IL-8 assists tumor growth through regulation of angiogenesis in the vascular endothelial systems. shRNA-induced HIF-1α and IL-8 knockdown inhibit angiogenesis and tumor growth in HCC

Therefore, simultaneous inhibition of HIF-1α and IL-8 expression has proven to be more effective in hindering angiogenesis than inhibition of a single factor [7, 32, 33]. With regards to expression at a molecular level, studies have demonstrated that liver cancer is regulated more by HIF-1α; however, in vascular endothelial cells, such as human umbilical vein endothelial cells (HUVEC), the level of IL-8 regulation is similar to that of HIF-1α [2, 41, 42]. The growth of cancer cells and VEGF expression, which controls angiogenesis, has been observed to be regulated by HIF-1α, whereas IL-8 does not affect tumor growth or VEGF expression [7]. Alternatively, in HUVEC, when IL-8 expression is inhibited, angiogenic inhibition is observed at a level similar to HIF-1α inhibition (Fig. 1).

Similar results have been obtained through animal research; inhibition of HIF-1α expression rarely resulted in tumors reproduction in the animal models. Moreover, no apoptosis of existing tumors are observed in these animals; however, in other animal models, where IL-8 expression was inhibited, a tumor volume similar to the time when shIL-8 was injected into the tumors was observed. These findings did not reveal any correlation between IL-8 and direct growth of tumors; however, IL-8 plays an important role in angiogenesis [24, 41, 43]. In addition, the results of the experiments on angiogenesis, such as invasion, tube formation, and aorta sprouting assays, have confirmed that simultaneous inhibition of two factors yielded more favorable responses than inhibition of a single factor [7]. Experiments with animal models have also demonstrated apoptosis of existing tumors as well as high survival rates in a majority of animals in which both the factors were inhibited. Moreover, it was confirmed that various factors to test for blood vessel formation, such as CD31, CD34, and vascular endothelial (V-E) cadherin, were not observed [7]. These findings suggested that controlling hypoxia as well as the expression of angiogenesis-associated factors that act via different pathways can aid in the inhibition of angiogenesis.

Various treatments have been developed for cancer, and better therapies have been developed by overcoming the limitations of already-developed treatments. We hypothesized that if the symptoms that occur during tumor treatment can be studied and controlled, the obstacles currently encountered during cancer treatment can be eliminated. If simultaneous regulation of tumor development, hypoxia, and angiogenesis is possible, cancer cells could be easily treated without peripheral damage. In other words, simultaneous inhibition of the factors that potentially control hypoxia and angiogenesis during treatment to induce apoptosis may be a more innovative anticancer treatment modality.

### Tumor escape from apoptosis under hypoxic conditions

A correlation exists between cancer and hypoxia [31]. Hypoxia during tumor development can destroy cancer cells; however, it acts as a key factor in excessive cancer proliferation [31, 44, 45]. Prevention of cancer via hypoxia treatment can be used as a highly effective anticancer therapy [2, 34, 46]. To date, studies have been carried out to induce apoptosis in various tumors [2, 34, 44, 47]. One potential treatment option involves reducing angiogenesis, typically by inhibiting VEGF, EGF, or bFGF, while an alternative option involves activating the intracellular intrinsic apoptosis pathway by inducing the expression of apoptotic factors and inhibiting the expression of anti-apoptosis factors [31]. Moreover, a method to stimulate an extracellular death signal in order to induce apoptosis could also be developed. Apoptosis, also referred to as programmed cell death, is one of the most important cellular functions [48]. In normal cells, a decrease in telomere length normally occurs with age; however, DNA damage, toxin exposure, and deprivation of growth factor also generate death signals in various pathways, which results in apoptosis [49, 50]. Hypoxic stimulation is also a crucial death signal; apoptosis is induced when the oxygen supply, required for the production of ATP, an important cellular metabolite, is suppressed [31]. However, in tumors, stimulation that induces apoptosis can be avoided [51]. During telomere length decrease, production of telomerase is promoted to restore the length of telomeres, and when DNA damage is induced, it can be repaired by mutation [31, 34, 52]. In hypoxic conditions, apoptosis can be avoided by inducing angiogenesis while increasing the expression of growth factors, thereby restoring oxygen supply, or by stimulating intracellular nitric oxide synthase (iNOs) through hematopoiesis and local vasodilation (Table 2) [6, 11, 53]. Apoptosis can be also be avoided by increasing anaerobic ATP production via glycolysis, which is facilitated by promoting GLUT1 or pyruvate dehydrogenase kinase activity [11].

**Table 2:** Role of anti-apoptosis under hypoxia

Factors	Functions	Ref
Bcl family	Block BAX and BAD activity	[11, 51]
	Inhibited caspase activation	[5, 44]
	Inhibited cytochrome c release	[31, 48]
BNIP	Apoptotic protector	[15]
TGF-alpha	Induces epithelial development	[26]
	Initiate multiple cell proliferation	[5, 44]
Caspase family	Downreulgation of caspase-3 and 9	[31, 48]
JNK	Regulates cell growth, differentiation, survival	[18, 51]
	up-regulated STAT3	[44]

HIF-1α, which is generated under hypoxic conditions, is an important anti-apoptotic factor [31, 45]. HIF-1, like tumor necrosis factor (TNF)-a, activates the expression of FoxM1, that in turn induces growth of cancer cells in the liver and increases resistance to apoptosis [50]. The expression of HIF-1 in liver cancer inhibits expression of various caspases and reduces expression of Bax and Bak, which lead to a higher concentration of cytochrome C inside the cells [32]. Increase in the expression of survivin and the Bcl-family, which are important factors that cause DNA fragmentation, can prevent hypoxic apoptosis [54].

**Fig. 2:**
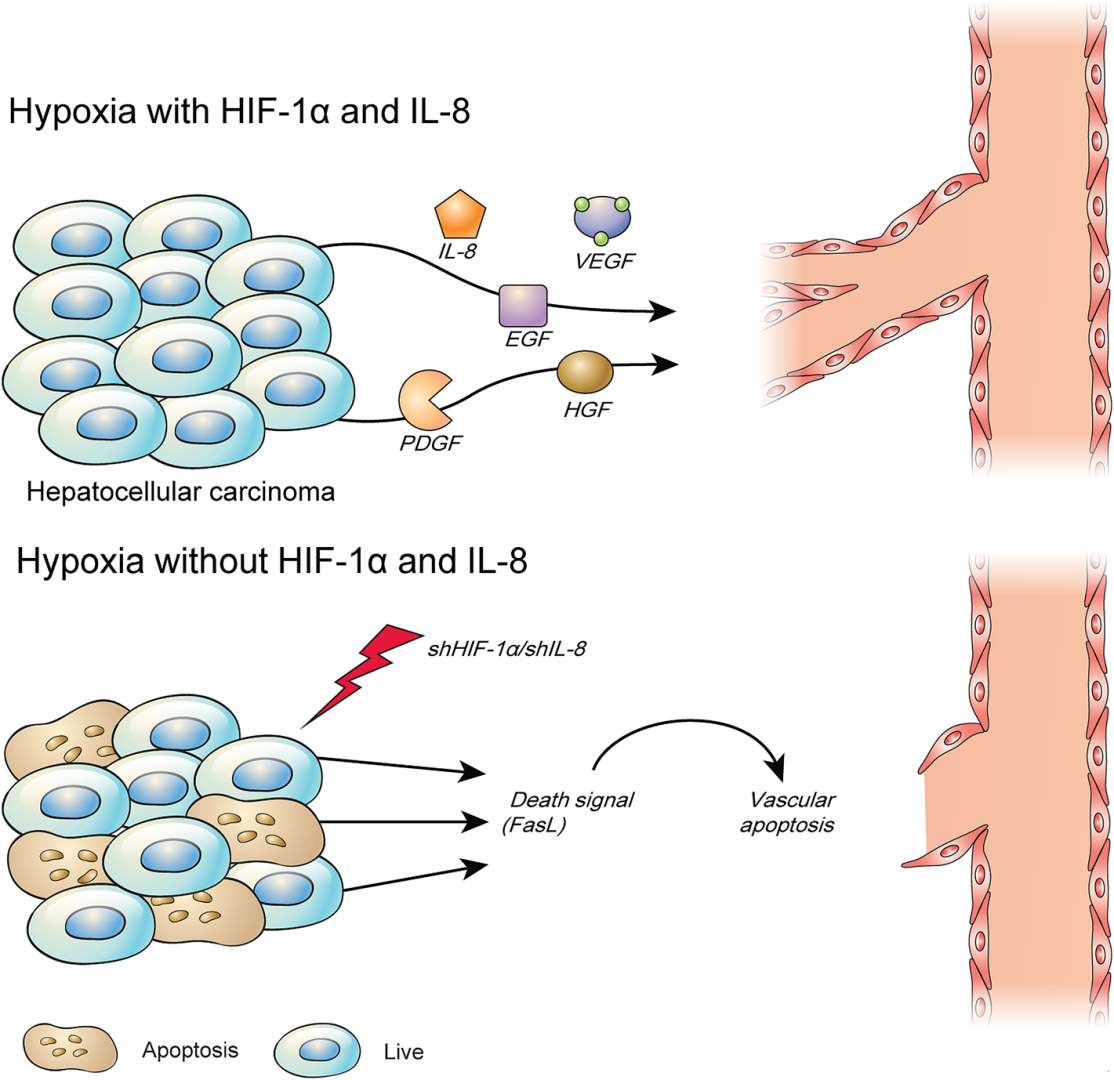
Regulation of hypoxic apoptosis in hepatocellular carcinoma. Apoptosis is an important mechanism for the development of organisms. Organisms survive and proliferate in the cyclic structure of cell creation and death. However, apoptosis is critical for inhibiting the growth of cancer cells. One of the significant survival mechanisms of cancer cells is the suppression or prevention of apoptosis. Adenovirus-mediated knockdown of HIF-1α and IL-8 induced apoptosis in HCC and triggered apoptosis of vascular endothelial cells

Apoptosis in tumors is important because tumor angiogenesis, which is increased by tumor proliferation, can also be inhibited [34]. Apoptosis in tumors can also induce apoptosis in newly formed peripheral blood vessels, thereby ensuring the prevention of a relapse of cancer or cancer stem cell growth at an early stage [1, 27, 28]. The immunofluorescence-TUNEL technique has demonstrated that tumors in which HIF-1α expression is inhibited display increased DNA fragmentation [32]. Interestingly, although IL-8 does not exert a direct influence on tumor apoptosis, it plays a role in tumor apoptosis by controlling apoptosis in blood vessels [32]. Cultivated tumor cell lines with simultaneous inhibition of these two factors demonstrated increase in tumor apoptosis via the FACS-TUNEL technique. It was also confirmed that a vascular endothelial cell culture medium developed from a culture medium, in which apoptosis had been induced in tumor cells, promoted apoptosis in vascular endothelial cells without any stimulation (Fig. 2) [3, 32]. Apoptosis in tumors affects the surrounding tissues owing to the constant communication and transmission between cells. The blood vessels, which are essential for tumor growth, mutually communicate via various factors that are present in the vicinity of the tumor. Therefore, cancer treatment and anticancer drugs can induce apoptosis in tumors while simultaneously regulating the expression of an activation factor in vascular endothelial cells, a higher anti-tumor therapeutic efficacy can be achieved during treatment of tumors [3, 32, 37]. In addition, a more precise tumor treatment can be developed by eliminating the factors that support the growth of malignant tumors, which relapse due to various reasons after tumor treatment [27, 32].

Tumors can be treated using various methods and with several drugs [1]; the efficacy of these treatments can confirmed via different experiments. Moreover, various studies have been conducted to develop potential anti-tumor treatments that work by regulating the microenvironment of the tumor or controlling various tumor growth factors along with the existing tumor treatments [1, 22, 28, 39, 40]. Inhibition of the hypoxic mediator, HIF-1α, and the activation factor in V-E cells, IL-8, which is closely related to tumor development, can potentially be used to develop a treatment that can directly regulate tumor development as well as the microenvironment of tumors [7, 14, 20, 32].

## Conclusion

Although newly developed treatments for HCC employ various approaches to combat the disease, all are associated with significant side effects and complications [1]. For example, in TACE, which uses vessel embolization to induce cancer tissue necrosis, surrounding tissue is also obliterated [30]. Furthermore, any remaining embolization- or radiotherapy-resistant cancer tissue tends to be more malignant and can lead to metastasis [55, 56]. Additionally, hypoxia induced by medical or surgical treatment induces the accumulation of HIF-1α inside tumor cells and its subsequent migration into the nuclei, where it promotes the expression of angiogenesis-related genes and increases oxygen supply to the tumor [57]. It also induces the expression of metastasis-related genes [12, 17, 31]. These hypoxia-induced processes reduce cellular injury and enable continuous tumor growth by ensuring an effective supply of oxygen to the tumor [31].

In recent studies, the inhibition of HIF-1α expression failed to block angiogenesis induced by the tumor, allowing the tumor to survive and proliferate [7, 27]. The key factor involved in this process is IL-8, which is up-regulated by hypoxic conditions during tumor proliferation [12, 56, 58]. IL-8 induces angiogenesis by activating vascular endothelial cells [59]. HIF-1α directly regulates HCC development and IL-8 assisted tumor growth through regulation of angiogenesis in the vascular endothelial system [28] s. These findings might be used as a basis for the development of an effective treatment that does not harm normal cells. However, further studies must be conducted before any clinical application. Although the inhibition of HIF-1α and IL-8 has been found to have a significant influence on tumor angiogenesis in animal studies, the effect thereof was restricted to specific hypoxic conditions. Since hypoxia destroys both tumor cells and normal cells, the expression of HIF-1α must be maintained in normal tissues.

Regarding tumor proliferation, a hypoxic state is important for tumor growth to start. It is thought that HIF-1 expression (HIF-1α and HIF-1β) controls the initiation of tumor growth and can affect anti-tumor growth by changing growth to be more malignant in a hypoxic state. Further study is required to determine other possible functions of HIF-1β that are comparatively less known than those of HIF-1α, which has drawn most of the attention until now.

A local hypoxic microenvironment is one of the most important characteristics of solid tumors. Apoptosis is an important mechanism for the development of organisms, which survive and proliferate in the cyclic structure of cell creation and death. However, apoptosis is also critical to inhibiting the growth of cancer cells. One of the significant survival mechanisms of cancer cells is the suppression or prevention of apoptosis. Once apoptosis is induced, cancer cells induce expression of various anti-apoptotic factors, thereby suppressing apoptosis. Thereby, the growth of cancer cells and tissues increases. Various anticancer agents or anticancer therapies have been rapidly developed to address this characteristic. TACE, which is currently widely utilized in the treatment of liver cancer, induces hypoxia and hypoglycemia in liver cancer cells and reduces the numbers of cancer cells. Radiation therapies also induce an extended range of hypoxia in radiated areas, thereby promoting hypoxic apoptosis of the tissues. Among the various treatments of liver cancer, most methods induce apoptosis of the cancer cells, thereby killing them.

The present study investigated the effects of cancer-cell induction or prevention of apoptosis on peripheral vascular cells, rather than the direct treatment of cancer cells [34, 40, 48]. Apoptosis of cancer cells was confirmed to influence the apoptosis or growth of peripheral tissues through various experiments related to apoptosis. Moreover, RNA expression was found to be regulated by a variable knockdown mechanism of RNA interference tools in vitro and in vivo [60]. The in vivo analysis easily used adenovirus-mediated shRNA for effective knockdown of target genes and directly-injected tumor tissues. The in vitro analysis also used lentiviral-mediated siRNA for effective knockdown of target genes in a rapid-growth cell-based assay [60, 61].

Various treatments have been developed for cancer, and better therapies have been developed by overcoming the limitations of already developed treatments. We hypothesized that if the symptoms that occur during tumor treatment can be studied and controlled, the obstacles currently encountered during cancer treatment can be eliminated. If simultaneous regulation of tumor development, hypoxia, and angiogenesis is possible, cancer cells could be easily treated without peripheral damage. In other words, simultaneous inhibition of the factors that potentially control hypoxia and angiogenesis during treatment to induce apoptosis may be a more innovative anticancer treatment modality.

## Abbreviations

RNAi: ribonucleic acid interference
HCC: hepatocellular carcinoma
HIFs: hypoxia inducible factors
IL-8: interleukin-8
HB(C)V: hepatitis B(C) virus
HUVECs: human umbilical vein endothelial cells
FACS-TUNEL: flow cytometry-terminal deoxynucleotidyl transferase dUTP nick end labeling
TACE: transcatheter arterial chemoembolization
VEGF: vescular endothelial growth factor
EGF: endothelial growth factors
bFGF: basic fibroblast growth factor
