# Erratum to: Regulation of the hypoxic tumor environment in hepatocellular carcinoma using RNA interference

**DOI:** 10.1186/s12935-017-0438-2

**Published:** 2017-07-12

**Authors:** Sung Hoon Choi, Jun Yong Park

**Affiliations:** 10000 0000 9149 5707grid.410885.0Division of Bioconvergence Analysis, Drug & Disease Target group, Korea Basic Science Institute, 169-148 Gwahak-ro, Yuseong-gu, Daejeon, 34133 Republic of Korea; 2grid.413046.4Yonsei Liver Center, Yonsei University Health System, Seoul, South Korea; 30000 0004 0470 5454grid.15444.30Department of Internal Medicine, Institute of Gastroenterology, Yonsei University College of Medicine, 50 Yonsei-ro, Seodaemun-gu, Seoul, 120-752 South Korea; 40000 0004 0470 5454grid.15444.30Institute of Gastroenterology, Yonsei University College of Medicine, Seoul, South Korea

## Erratum to: Cancer Cell Int (2017) 17:3 DOI 10.1186/s12935-016-0374-6

After the publication of the original article [1], it was noted that the pre-revision version of this manuscript was mistakenly published. This erratum contains the correct, revised edition of this manuscript.

## Abstract

Hypoxia, which arises in tumor cells that have been deprived of oxygen, has been shown to play a role in tumor development in hepatocellular carcinoma. Hypoxia-inducible factors (HIFs) are transcription factors that regulate cellular homeostatic responses to oxidative stress and have been identified as key transcriptional activators of tumor angiogenesis, survival, and metabolism. Cytokines, such as IL-8, also influence survival and angiogenesis in endothelial cells. IL-8 is overexpressed under hypoxic conditions and has been demonstrated to induce tumor angiogenesis and growth. Regulation of these oncological factors using RNA interference-based tools, small interfering RNA (siRNA) and short hairpin RNA (shRNA), were investigated in vitro and in vivo. The conclusion based on multiple studies is that regulation of HIFs and IL-8 by si/shRNA results in modulation of tumor angiogenesis and apoptosis in the tumor microenvironment. This review summarizes the results of studies investigating regulation of the hypoxic tumor environment.


**Keywords**


RNAi, HCC, HIF, IL-8, Angiogenesis, Apoptosis

## Background

Hepatocellular carcinoma (HCC) is the sixth most common, and the second most lethal, cancer in the world [1]. Approximately 80–90% of cirrhotic liver disease cases resulting from chronic viral hepatitis B or C develop into HCC [1]. Moreover, hypoxic induction of angiogenesis and tumor growth is observed in most cases of advanced HCC [2].

The most important factors that influence HCC progression are oxygen and nutrients [3]. The liver is an organ with a specific blood supply. Approximately 25 and 75% of the total blood volume entering the liver does so via the hepatic artery and the portal vein, respectively. The latter drains into structures of smaller diameter called sinusoids. Vascular resistance is very low in these structures, and portal venous blood, which is loaded with food and large numbers of microbial antigens from the intestine, flows extremely slowly into the sinusoids. Thus, large amounts of nutrients and oxygen are required for HCC cell proliferation, resulting in localized hypoxia [3, 4]. This hypoxic environment causes tumor angiogenesis, i.e., the generation of new blood vessels from existing ones [3, 5]. Tumor angiogenesis overcomes oxidative stress [6] and the deficiency in oxygen-dependent energy production caused by hypoxia [3, 7]. The key factors responsible for regulation of angiogenesis during hypoxia are hypoxia-inducible factor (HIF)-1α and vascular endothelial growth factor (VEGF) [5, 8]. However, various studies have reported that angiogenesis is induced even during inhibition of HIF-1α during hypoxia [9], demonstrating that tumor angiogenesis is partially affected by various other factors [9]. Increased expression of various factors, such as growth factors and tumor-stimulating factors, can induce angiogenesis during hypoxia, as they increase proliferation and ensure stabilization of endothelial cells. These factors are induced not only by HIF-1α, but also by cytokines such as interleukin (IL)-8 or by growth factors such as platelet-derived growth factor [10, 11]. In addition, these factors increase VEGF expression, as well as help increase and stabilize angiogenesis, by upregulating VEGF receptor on the surface of endothelial cells [12]. Moreover, various solid tumors pass through the following three stages during their reproductive cycle: cell proliferation, hypoxia, and recovery tumor survival by angiogenesis [3, 13, 14].

## Main text

### Roles of HIF and IL-8 under hypoxic conditions

The hypoxia inducible factors (HIFs) are a family of heterodimeric transcription factors that act as master regulators of the homeostatic transcriptional response to hypoxia in virtually all cells and tissues [3]. Active HIF consists of alpha and beta subunits [2, 15, 16]. Three alpha subunits, termed HIF-1α, HIF-2α, and HIF-3α, have been described in humans, mice, and rats; all bind to the common beta subunit HIF-1β, also known as aryl –hydrocarbon receptor nuclear translocator (ARNT) [17, 18]. Active HIF is named according to its alpha subunit; hence, HIF-1 consists of HIF-1α and ARNT, HIF-2 of HIF-2α and ARNT, and so forth [19]. HIF-1 and HIF-2 are the major hypoxia-inducible factors in humans, mice, and rats [17].

Under conditions of normoxia, HIF-1α subunits are hydroxylated at proline residues by hydroxylases [3, 20]. Hydroxylation of HIF-1α and assembly of a protein scaffold consisting of the von Hippel–Lindau (VHL) tumor suppressor [21–24], along with other co-factors, result in rapid ubiquitination of the alpha subunit and subsequent degradation by the proteasome. Conversely, under conditions of hypoxia, HIFα subunits escape degradation and are free to dimerize with their binding partner, ARNT [3, 17]. HIF translocates to the nucleus where it affects the transcription of target genes, typically by binding to a hypoxia response element (HRE) in the upstream promoter region of such target genes, which include genes related to angiogenesis, apoptosis, metabolism, and survival [2, 3, 18].

Tumor cells have the ability to exploit the expression of various cytokines and their receptors for their own use. Cytokines secreted by tumor cells can act on the surrounding normal stroma, such as blood vessels, recruiting them to aid in tumor growth, survival, and metastasis. IL-8/CXCL-8 is a pro-inflammatory cytokine [25] and a key molecule influencing endothelial cell survival and angiogenesis [26]. IL-8, which is a novel leukocyte chemotactic-activating cytokine [27, 28], is produced by various types of cells upon contact with inflammatory stimuli and exerts a variety of functions on leukocytes [29, 30], particularly neutrophils. IL-8 is also associated with several types of acute inflammatory reactions [25], including lipopolysaccharide (LPS)-induced dermatitis, LPS/IL-1-induced arthritis, and lung reperfusion injury [29]. IL-8 can promote resolution of infection by inducing phagocytosis, oxidative burst, and the release of DNA webs known as neutrophil extracellular traps that trap and kill invading microbes [27]. Conversely, IL-8 is also regulated under hypoxic conditions and directly regulates endothelial cells [31–34]. IL-8 has been shown to regulate pathological angiogenesis, tumor growth, and metastasis [33]. The mechanism(s) regulating IL-8-mediated endothelial cell survival are not well understood. Recent reports suggest that in addition to cell proliferation and migration, endothelial cell survival and death are also important factors for tumor survival and development [34]. Other studies have shown that a cell cycle-regulated apoptosis inhibitor, survivin, and the cell death-related gene products, Bcl-xL and Bcl-2 [19, 35], are associated with VEGF-induced angiogenesis [12, 34]. IL-8 and its receptors CXCR1 and CXCR2 have been shown to play a role in endothelial cell proliferation [34]. Liver cancers, such as HCC, are dependent on angiogenesis; therefore, inhibition of angiogenesis could be a potential treatment modality to prevent the proliferation and growth of solid tumors [36, 37]. Thus far, efforts to treat solid tumors using angiogenesis inhibitors have yielded good results [37, 38]. However, these therapies affect not only solid tumors but also normal cells, which is an area of concern in cancer treatment [36]. Furthermore, cancer therapies such as transarterial chemoembolization (TACE) delivered via blood vessels may not produce the desired effect and may even increase vascular proliferation and growth of malignant tumors by incomplete responses of TACE therapy [39]. Correlations among hypoxia, cancer proliferation, angiogenesis, and tumor growth or development have been observed [40]. Elucidation of the relationships among these processes may ascertain the basis for inhibition of tumor growth and metastasis. Management of the tumor can be achieved by dual control of HIF-1α and angiogenic factors [8]. Innovative and more effective cancer therapies can be developed by regulating the expression of HIF-1α, which is the key factor in hypoxia, and by controlling the expression of IL-8 and other angiogenic stimulators, which restore the angiogenic processes caused by inhibition of HIF-1α expression [9].

HIF-1α knockdown directly represses tumor growth, whereas IL-8 knockdown indirectly represses tumor growth [1, 9, 36]. Combined knockdown of HIF-1α and IL-8 increased survival rates in mice [9]. Conditioned media collected from HCC cells subjected to combined knockdown also decreased microvessel density and tumor volume in vivo [9]. Similarly, combined knockdown of HIF-1α and IL-8 inhibited the angiogenic effects of HCC cell-conditioned media on tube formation and invasion by endothelial cells in vitro [9]. Inhibition of HIF-1α and IL-8 upregulated the expression of apoptotic factors while simultaneously downregulating the expression of anti-apoptotic factors [9]. Knockdown of HIF-1α and IL-8 increased the concentration of cytosolic cytochrome C and enhanced DNA fragmentation in HCC cell lines and human umbilical vein endothelial cells (HUVECs) [41]. Moreover, apoptosis was induced in HUVECs treated with culture supernatant collected from HCC cell lines with silenced HIF-1α and IL-8 expression, under conditions of hypoxia [41]. Silencing of HIF-1β expression suppressed tumor cell growth and inhibited the expression of tumor growth-related factors [42], such as VEGF, epidermal growth factor (EGF), and hepatocyte growth factor. Suppression of tumor cell invasion and migration was also demonstrated in HIF-1β-silenced HCC cell lines [42].

### Comparing various RNA silencing methods

The ability of RNA interference (RNAi) or transient gene ‘knockdown’ to silence target genes with high efficiency and specificity has stimulated efforts to develop these molecules into therapeutic agents [43]. RNAi can be classified into three types [44, 45], all of which have similar mechanisms of action. Firstly, long double-stranded RNAs (dsRNAs), approximately 500–1000 nucleotides in length, have been employed to evaluate gene functions [46]. Following introduction of exogenous dsRNA into the cytoplasm of cells [47], it gets cleaved by the RNase III enzyme Dicer to produce a short dsRNA called small interfering RNA (siRNA) [48]. The siRNA, which is 21–23 nucleotides in length, is loaded into a protein complex called the RNA-induced silencing complex (RISC) [47, 49]. The siRNA is then unwound and its sense strand degraded, while the surviving antisense strand guides the activated RISC to the target mRNA through full complementary binding [50]. The mRNA is then cleaved by RISC, leading to silencing of the target gene [51]. Since long dsRNA triggers an immunostimulatory response through the activation of Dicer-related antiviral pathways and induction of type 1 interferon [46, 52], it is less suitable for therapeutic use. In contrast, synthetic siRNA is a more promising gene silencing mediator, because it poses less risk of an immune reaction [53]. Based on this principle, siRNA therapies have been investigated extensively in preclinical studies [43, 44, 48], and some siRNA agents have already been investigated in clinical trials for the treatment of cancer and several other diseases [43]. To date, eight clinical trials of siRNA/dsRNA therapies for cancer have been reported [52, 54]. Various siRNA targets or pathways were evaluated in these trials, including polo-like kinase 1 [55, 56], KRAS (G12D) [57], protein kinase N3 [58], and VEGF [59, 60]. In addition, preliminary results have been released from a phase I trial of the first siRNA-based therapy targeting the oncogene MYC in patients with advanced solid tumors [43, 52].

Short hairpin RNAs (shRNAs) are delivered into cells using a DNA vector and then transcribed by either RNA polymerase II or III in the nucleus [45]. The primary transcript is called primary shRNA (pri-shRNA), which contains a hairpin-like stem-loop structure [47]. Pri-shRNA is processed into a 50–70-nucleotide-long loop-stem precursor shRNA (pre-shRNA) by a protein complex containing the RNase III nuclease Drosha and the dsRNA binding domain protein DGCR8 [49]. It is then transported to the cytoplasm by a specialized nuclear membrane protein, exportin 5 (Exp5) [47]. The loop sequence of the pre-shRNA is cleaved by Dicer to form a double-stranded siRNA [44, 47]. This endogenously produced siRNA is loaded into RISC and can induce RNAi through a similar process as the synthetic siRNA [47, 51]. Since shRNA expression units can be incorporated into viral vectors and continuously synthesized by host cells, shRNAs can induce long-lasting gene silencing effects [45, 47]. The RISC-loading process of shRNA is approximately 5- to 10-fold more efficient than that of siRNA, indicating that a lower dose of shRNA is required to maintain therapeutic efficacy with less off-target effects [45]. However, the shRNA approach is a DNA-based strategy depending on the expression of shRNA-encoding genes, which often require viral vectors (such as adenoviral or lentiviral vectors) [47, 51, 61]. From a delivery perspective, the introduction of synthetic siRNA into the cytoplasm is a more straightforward method to induce RNAi [61]. The use of viral vectors for delivery poses safety concerns in therapeutic applications [47, 61].

MicroRNAs (miRNAs) is a naturally occurring non-coding RNA molecules that play a key role in regulating gene expression [50]. Primary miRNAs (pri-miRNAs) are transcribed by RNA polymerase II from endogenous miRNA genes in the nucleus [62]. The hairpin-containing pri-miRNA is structurally similar to the pri-shRNA; therefore, miRNAs also have a similar function to shRNAs [45, 50]. The pri-mRNA is converted by the Drosha/DCGR8 complex into precursor miRNA (pre-miRNA), which consists of 70–100 nucleotides with interspersed mismatches, and adopts a loop structure [45, 63]. Pre-miRNA is subsequently transported to the cytoplasm by Exp5 and processed by Dicer into a mature miRNA of 18–25 nucleotides in length [62]. In contrast to siRNA, the antisense strand of miRNA is only partially complementary to the target mRNA, leading to gene silencing via translational repression and/or mRNA deadenylation [50]. Positions 2–7 at the 5′ end of miRNA is an essential sequence for target recognition, and the miRNA-binding sites of mRNA are located in the 3′ untranslated region [50, 63]. There are two major approaches in miRNA-based therapeutics: miRNA inhibition [64–66] and miRNA replacement [44, 67]. The former suppresses the activity of endogenous miRNA using an antisense oligonucleotide (anti-miR), and the latter introduces a synthetic miRNA (miRNA mimic) to restore the functions of endogenous miRNA [44, 49, 53, 67].

### Inhibition of HIF-1α and IL-8 expression as a strategy for suppression of angiogenesis

Angiogenesis is essential for tumor growth and metastasis, and attempts to control tumor-associated angiogenesis may prove to be promising tactics for limiting tumor progression [36]. Angiogenesis occurs during development and vascular remodeling as a controlled series of events leading to neovascularization, which supports changes in tissue requirements [68]. Blood vessels and stromal components are responsive to pro- and anti-angiogenic factors that allow vascular remodeling during development, wound healing, and pregnancy [69, 70]. However, in pathological situations, such as cancer, the same angiogenic signaling pathways are induced and exploited.

Although an oncogenic event may allow tumor cells to evade surveillance or may enhance their survival, large-scale growth of a tumor ultimately requires a blood supply [36]. To obtain this blood supply, tumor cells can tilt the balance toward the production of stimulatory angiogenic factors to drive vascular growth by attracting and activating cells from within the microenvironment of the tumor [71]. The magnitude and quality of the angiogenic response are ultimately determined by the sum of pro- and anti-angiogenic signals (Table 1) and, more specifically, their unique effects on multiple cell types [72]. Understanding how these various components are regulated is required for the design and development of effective anti-angiogenic therapies for cancer [36].

**Table 1 Tab1:** Up regulation of tumor angiogenic factors under hypoxia

Factors	Functions	References
VEGF	Increased vascular permeability	[5]
	Endothelial sprouting	[36]
	EC proliferation and migration	[10, 35]
	EC assembly	[40]
iNO	Increased vascular permeability	[35]
Angiopoietin-2	Endothelial sprouting	[36]
PDGF	Pericyte recruiting	[9, 56]
	EC proliferation and migration	[3, 40]
MMPs	Degradation of extracellular matrix	[5]
Tie2	Form the blood vessels	[40]
TGF-beta	Increased EC differentiation formation of vascular structure	[35, 40]
	Formed vascular structure	[10, 40]
VEGFR2	Stimulate endothelial cell mitogenesis and cell migration	[3, 10]
	Enhances microvascular permeability	[5, 56]
Endothelin	Regulates local vascular tone and integrity	[10, 40]
	Influences EC growth and survival	[5]
IL-8	Increased vascular permeability	[5]
	EC proliferation and migration	[25]
	Endothelial sprouting	[36]
Thrombospondin	Antiangiogenic, inhibiting the proliferation and migration of endothelial cells	[95, 96]

In cancer, multiple sources and modes of vascular remodeling contribute to disease progression [37]. Targeting one aspect of this remodeling process may produce a short-term effect; nevertheless, suppression of a particular pathway could result in the promotion of another [9]. The redundancy and diversity by which blood vessels can remodel might account for the poor efficacy or acquired resistance often observed in response to antiangiogenic therapies [3]. Improving therapeutic responses thus requires consideration of the signaling pathways that regulate the multiple cell types comprising the vascular components of cancer [73]. Once a tumor lesion exceeds a few millimeters in diameter, hypoxia and nutrient deprivation triggers an “angiogenic switch” to allow tumor progression [3, 19, 36]. Tumor cells exploit their microenvironment by releasing cytokines and growth factors to activate surrounding normal, quiescent cells, initiating a cascade of events that quickly becomes dysregulated [74].

Therefore, simultaneous inhibition of HIF-1α and IL-8 expression has proven to be more effective for hindering angiogenesis than has inhibition of a single factor [9, 41, 42]. With regard to molecular expression, studies have demonstrated that liver cancer under hypoxia is more highly regulated by HIF-1α; however, in vascular endothelial cells, such as HUVECs, the level of IL-8 regulation of angiogenesis is similar to that of HIF-1α [2, 75, 76]. In cancer cells, both VEGF expression, which controls angiogenesis, and cell growth appear to be regulated by HIF-1α, whereas IL-8 does not affect tumor growth or VEGF expression [9]. Alternatively, in HUVECs, IL-8 expression inhibition induced a similar level of angiogenic inhibition as that induced by HIF-1α inhibition (Fig. 1).

**Fig. 1 Fig1:**
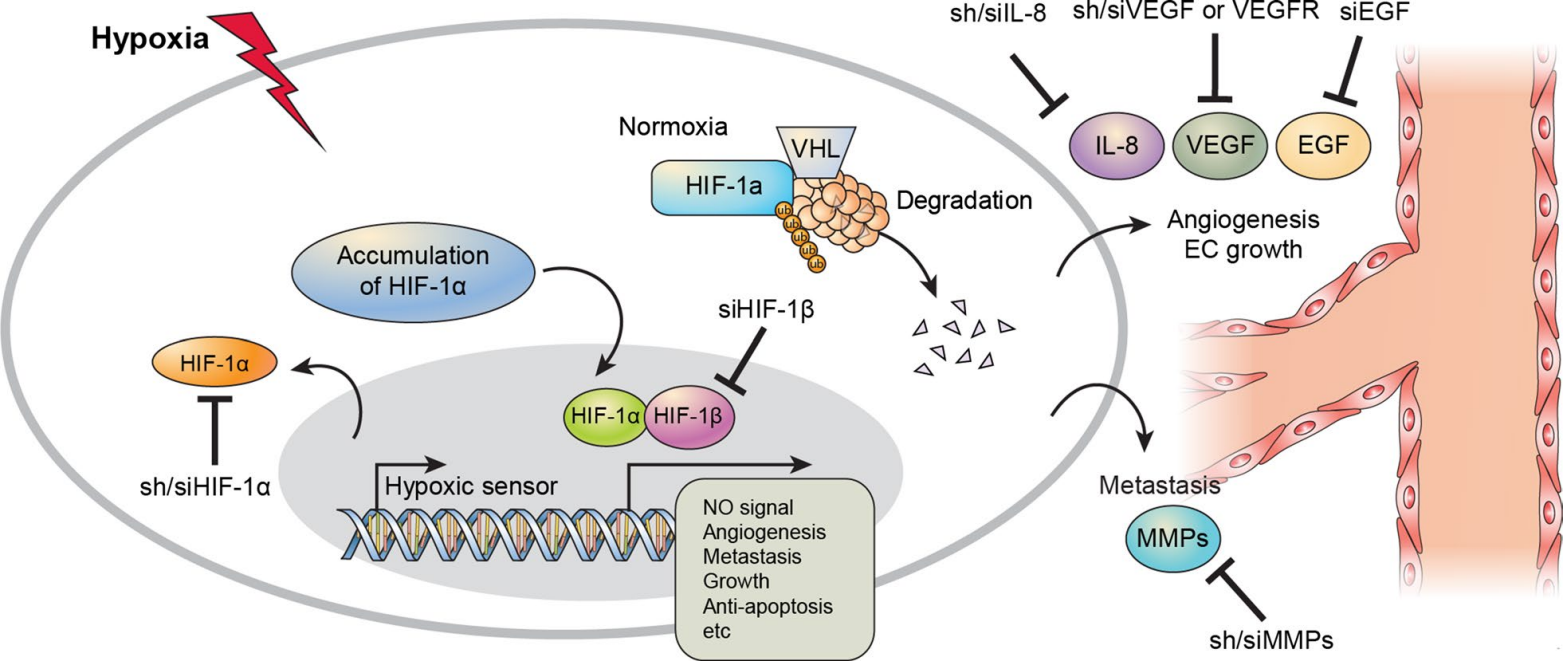
Inhibition of tumor angiogenesis by silencing of HIF-1α and IL-8. HIF-1α directly regulates HCC development and IL-8 assists tumor growth through regulation of angiogenesis in the vascular endothelial systems. shRNA-induced HIF-1α and IL-8 knockdown inhibit angiogenesis and tumor growth in HCC. And, variable si/shRNA used regulation of target gene, such as siVEGF, siMMPs, siEGFR, et al.

Similar results have been obtained through animal studies; inhibition of HIF-1α expression rarely results in tumor reproduction in animal models. Moreover, apoptosis was not detected in existing tumors in these animals. However, in other animal models in which IL-8 expression was inhibited, tumor volumes were similar to those in other experiments, in which a shRNA targeting IL-8 was injected into tumors. These findings did not reveal any direct correlation between IL-8 function and tumor growth; however, IL-8 plays an important role in angiogenesis [33, 75, 77]. In addition, the results of experiments investigating angiogenesis, such as invasion, tube formation, and aorta sprouting assays, confirmed that simultaneous inhibition of two factors yields more favorable responses than does inhibition of a single factor [9]. Experiments in animal models have also demonstrated apoptosis of existing tumors as well as high survival rates in the majority of animals in which both factors were inhibited. Moreover, various markers of blood vessel formation, such as CD31, CD34, and vascular endothelial-cadherin, were not observed [9]. These findings suggest that in addition to controlling hypoxia, targeting the expression of angiogenesis-associated factors that act via different pathways can help inhibit angiogenesis.

Improved cancer therapies have been developed by overcoming the limitations of existing therapies. We hypothesize that if the symptoms associated with cancer treatment can be monitored and controlled, certain obstacles currently encountered during treatment can be eliminated. If it were possible to regulate tumor development, hypoxia, and angiogenesis simultaneously, cancer cells could be treated easily without peripheral damage. In other words, delivery of therapies that simultaneously inhibit factors controlling hypoxia and angiogenesis, while concurrently inducing apoptosis, may represent a more innovative anticancer treatment modality than those currently available.

### Tumor escape from apoptosis under hypoxic conditions

The relationship between cancer and hypoxia is paradoxical; while hypoxia during tumor development can destroy cancer cells, it also acts to regulate excessive cancer proliferation [40, 78, 79]. Induction of hypoxia has been reported to be a highly effective anticancer therapy [2, 68, 80]. Several studies have been performed to assess the effects of inducing apoptosis in various tumors [2, 68, 78, 81]. One potential treatment option reduces angiogenesis, typically by inhibiting VEGF, EGF, or basic fibroblast growth factor, while an alternative option involves activating the intracellular intrinsic apoptosis pathway by inducing the expression of apoptotic factors and inhibiting the expression of anti-apoptotic factors [40]. An additional approach could be stimulation of an extracellular death signal to induce apoptosis. Apoptosis, also referred to as programmed cell death, is one of the most important functions of a cell [82]. In normal cells, a decrease in telomere length normally occurs with age; however, DNA damage, toxin exposure, and deprivation of growth factors also generate death signals resulting in apoptosis [83, 84]. Hypoxic stimulation is also a crucial death signal; apoptosis is induced when the oxygen supply, which is required for the production of ATP, an important cellular metabolite, is suppressed [40]. However, tumors can develop mechanisms to avoid responding to apoptosis-inducing signals [85]. Upon reduction of telomere length, telomerase production is promoted to restore telomere length, and when DNA damage is induced, it can be repaired by mutation [40, 68, 86]. Under hypoxic conditions, apoptosis can be avoided by inducing angiogenesis while increasing the expression of growth factors, thereby restoring oxygen supply, or by stimulating the production of intracellular nitric oxide synthase via hematopoiesis and local vasodilation (Table 2) [8, 15, 87]. Apoptosis can be also be avoided by increasing anaerobic ATP production via glycolysis, by promoting GLUT1 or pyruvate dehydrogenase kinase activity [15].

**Table 2 Tab2:** Role of anti-apoptosis under hypoxia

Factors	Functions	References
Bcl family	Block BAX and BAD activity	[11, 51]
	Inhibited caspase activation	[5, 44]
	Inhibited cytochrome c release	[31, 48]
BNIP	Apoptotic protector	[15]
TGF-alpha	Induces epithelial development	[26]
	Initiate multiple cell proliferation	[5, 44]
Caspase family	Downregulation of caspase-3 and 9	[31, 48]
JNK	Regulates cell growth, differentiation, survival	[18, 51]
	Up-regulated STAT3	[44]

HIF-1α, which is generated under hypoxic conditions, is an important anti-apoptotic factor [40, 79]. HIF-1, like tumor necrosis factor-α, activates the expression of FoxM1, which induces cancer cell growth in the liver and increases resistance to apoptosis [84]. The expression of HIF-1 in liver cancer inhibits the expression of various caspases and reduces the expression of Bax and Bak, thereby leading to a higher intracellular concentration of cytochrome C [41]. Increased expression of survivin and Bcl family members, which are important factors causing DNA fragmentation, can prevent hypoxia-induced apoptosis [88].

Apoptosis in tumors is important because it can inhibit tumor angiogenesis, which is increased by tumor proliferation [68], and can induce apoptosis in newly formed peripheral blood vessels, thereby preventing relapsed growth of cancer cells or cancer stem cells at an early stage [1, 36, 37]. The immunofluorescence terminal deoxynucleotidyl transferase dUTP nick end labeling (TUNEL) technique has been used to demonstrate that tumors in which HIF-1α expression has been inhibited display increased DNA fragmentation [41]. Interestingly, although IL-8 does not exert a direct influence on tumor apoptosis, it regulates apoptosis in blood vessels [41]. Cultured tumor cell lines in which both of these factors, such as HIF and IL-8, were inhibited simultaneously demonstrated an increase in tumor apoptosis via the Fluorescence-activated cell sorting(FACS)-TUNEL technique. It was also observed that cell culture medium from tumor cells induced to undergo apoptosis promoted apoptosis in vascular endothelial cell cultures without the need for any stimulation (Fig. 2) [3, 41]. Apoptosis in tumors affects surrounding tissues because of constant communication and signal transmission between cells. Blood vessels, which are essential for tumor growth, communicate via various factors present in the tumor vicinity. Therefore, if anticancer drugs could induce apoptosis in tumors while simultaneously regulating the expression of certain activated factors in vascular endothelial cells, a superior therapeutic efficacy could be achieved in tumors [3, 41, 71]. In addition, more targeted tumor treatments could be developed by eliminating factors that support the growth of malignant tumors, which can relapse following treatment [36, 41].

**Fig. 2 Fig2:**
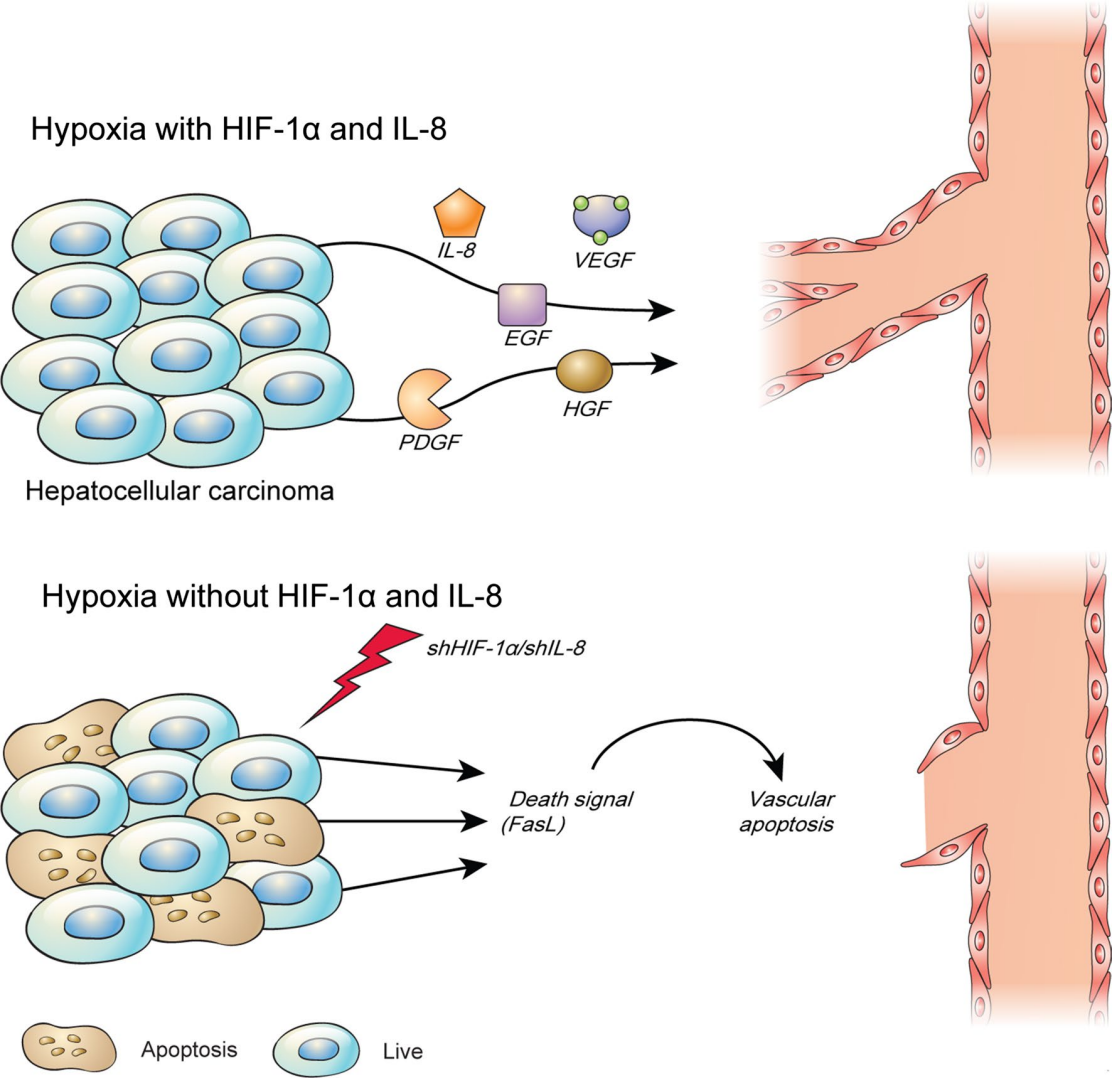
Regulation of hypoxic apoptosis in hepatocellular carcinoma. Apoptosis is an important mechanism for the development of organisms. Organisms survive and proliferate in the cyclic structure of cell creation and death. However, apoptosis is critical for inhibiting the growth of cancer cells. One of the significant survival mechanisms of cancer cells is the suppression or prevention of apoptosis. Adenovirus-mediated knockdown of HIF-1α and IL-8 induced apoptosis in HCC and triggered apoptosis of vascular endothelial cells

Tumors can be treated by various methods and several drugs [1]; the efficacy of these treatments has been confirmed via different experiments. Moreover, various studies have been conducted to develop potential anti-tumor treatments that regulate the tumor microenvironment or modulate various tumor growth factors as well as target the tumor itself [1, 31, 37, 73, 74]. Methods that inhibit the hypoxic mediator HIF-1α and the vascular endothelial cell activation factor IL-8, which have been implicated in tumor development, could potentially be used to develop a treatment that could directly regulate both tumor development and the tumor microenvironment [9, 18, 24, 41].

## Conclusions

Although newly developed treatments for HCC employ various approaches to combat this disease, all are associated with significant side effects and complications [1]. For example, TACE, which embolizes vessels to induce cancer tissue necrosis, also causes damage to surrounding tissues [39]. Furthermore, any remaining embolization- or radiotherapy-resistant cancer tissue tends to be more malignant and can metastasize [89, 90]. Additionally, hypoxia induced by medical or surgical treatment induces the accumulation of HIF-1α inside tumor cells and its subsequent translocation into the nucleus, where it promotes the expression of angiogenesis-related genes and increases oxygen supply to the tumor [91]. It also activates the expression of metastasis-related genes [16, 21, 40]. These hypoxia-induced processes reduce cellular injury and enable continuous tumor growth by ensuring that the tumor receives an adequate supply of oxygen [40].

In recent studies, inhibition of HIF-1α expression failed to block tumor-induced angiogenesis, allowing the tumor to survive and proliferate [9, 36]. The key factor involved in this process is IL-8, which is upregulated in response to hypoxic conditions during tumor proliferation [16, 90, 92]. IL-8 induces angiogenesis by activating vascular endothelial cells [93]. HIF-1α directly regulates HCC development and IL-8-assisted tumor growth via regulation of angiogenesis in the vascular endothelial system [37]. These findings might aid the development of effective treatments that do not harm normal cells. However, further studies must be conducted before any clinical applications can be performed. Although inhibition of HIF-1α and IL-8 had a significant effect on tumor angiogenesis in animal studies, this effect was restricted to specific hypoxic conditions. Since hypoxia destroys both tumor and normal cells, the expression of HIF-1α must be maintained in normal tissues.

Regarding tumor proliferation, hypoxia is an important condition for the initial growth of a tumor. It is thought that HIF-1α and HIF-1β expression regulates the initiation of tumor growth and induces more malignant growth under hypoxic conditions. Further studies will be required to determine other possible functions of HIF-1β, which are comparatively less known than those of HIF-1α, which has been the focus of most investigations at this point.

A local hypoxic microenvironment is one of the most important characteristics of solid tumors. Apoptosis is a critical mechanism in the development of organisms. However, apoptosis is also critical for inhibition of cancer cell growth. A significant survival mechanism in cancer cells is the suppression or prevention of apoptosis. Once apoptosis is induced, cancer cells induce expression of various anti-apoptotic factors, thereby suppressing apoptosis and promoting increased growth of cancer cells and tissues. Various anticancer agents or therapies have been rapidly developed to address this characteristic. TACE, which is widely utilized for the treatment of liver cancer currently, induces hypoxia and hypoglycemia in liver cancer cells, reducing the number of cancer cells. Radiation therapies also induce extended hypoxia in radiated areas, thereby promoting hypoxia-induced apoptosis of cancer tissues. Among the various treatments for liver cancer, most kill cancer cells via apoptosis.

The present study investigated the effects of induction or prevention of apoptosis in peripheral vascular cells, rather than the direct treatment of cancer cells [68, 74, 82]. Apoptosis of cancer cells was confirmed to influence apoptosis or growth of peripheral tissues in various experiments investigating apoptosis. Moreover, RNA expression was found to be regulated by various knockdown mechanisms when employing RNAi tools in vitro and in vivo [47]. In vivo analyses used adenovirus-mediated shRNA directly injected into tumor tissues for effective knockdown of target genes. In vivo analyses also used lentivirus-mediated siRNA for effective knockdown of target genes in a rapid-growth cell-based assay [47, 94].

Improved cancer therapies have been developed by overcoming certain limitations associated with existing treatments. We hypothesized that if symptoms that occur during tumor treatment could be studied and controlled, obstacles that are currently encountered during cancer treatment could be eliminated. If simultaneous regulation of tumor development, hypoxia, and angiogenesis is possible, cancer cells could be easily treated without peripheral damage. In other words, a method that employs simultaneous inhibition of factors that potentially regulate hypoxia and angiogenesis while also inducing tumor apoptosis may represent a more innovative anticancer treatment modality than those currently available.

### Abbreviations

RNA: iribonucleic acid interference; siRNA: small interference RNA; shRNA: small hairpin RNA; HCC: hepatocellular carcinoma; HIFs: hypoxia inducible factors; IL-8: interleukin-8; HB(C)V: hepatitis B(C) virus; HUVECs: human umbilical vein endothelial cells; FACS-TUNEL: flow cytometry-terminal deoxynucleotidyl transferase dUTP nick end labeling; TACE: transcatheter arterial chemoembolization; VEGF: vascular endothelial growth factor; EGF: endothelial growth factors; bFGF: basic fibroblast growth factor

### Authors’ contributions

SHC contributed to analysis and interpretation of data and drafting of the manuscript. JYP contributed to study concept and design, confirmed and revised the manuscript for important intellectual content. Both authors read and approved the final manuscript.

### Acknowledgements

We are grateful to Dong-Su Jang (Medical Illustrator, Medical Research Support Section, Yonsei University.

### Competing interests

The authors declare that they have no competing interests.

### Availability of data and materials

Data sharing not applicable to this article as no datasets were generated or analyzed during the current study.

### Funding

This research was supported by the Basic Science Research Program through the National Research Foundation of Korea (NRF), funded by the Ministry of Education, Science, and Technology (NRF-2011-0014537).

## References


Gerard B, Bleiberg H. Treatment of hepatocarcinoma. Curr Oncol Rep 2004;6(3):184–91.Wong CC, Kai AK, Ng IO. The impact of hypoxia in hepatocellular carcinoma metastasis. Front Med 2014;8(1):33–41.Carmeliet P, Dor Y, Herbert JM, Fukumura D, Brusselmans K, Dewerchin M, et al. Role of HIF-1alpha in hypoxia-mediated apoptosis, cell proliferation and tumour angiogenesis. Nature 1998;394(6692):485–90.Alidoosti M, Ghaedi M, Soleimani A, Bakhtiyari S, Rezvanfard M, Golkhu S, et al. Study on the role of environmental parameters and HIF-1A gene polymorphism in coronary collateral formation among patients with ischemic heart disease. Clin Biochem 2011;44(17–18):1421–4.Feng L, Tao L, Dawei H, Xuliang L, Xiaodong L. HIF-1alpha expression correlates with cellular apoptosis, angiogenesis and clinical prognosis in rectal carcinoma. Pathol Oncol Res 2014;20(3):603–10.Caraglia M, Giuberti G, Marra M, Addeo R, Montella L, Murolo M, et al. Oxidative stress and ERK1/2 phosphorylation as predictors of outcome in hepatocellular carcinoma patients treated with sorafenib plus octreotide LAR. Cell Death Dis 2011;2:e150.Marra M, Sordelli IM, Lombardi A, Lamberti M, Tarantino L, Giudice A, et al. Molecular targets and oxidative stress biomarkers in hepatocellular carcinoma: an overview. J Transl Med 2011;9:171.Hao LS, Wang G, Qian K, Luo T, Li XJ, Wu XT. HIF-1alpha expression and relationship involving tumor cell proliferation and angiogenesis in human breast carcinoma. Sichuan Da Xue Xue Bao Yi Xue Ban 2007;38(1):60–3.Choi SH, Kwon OJ, Park JY, Kim do Y, Ahn SH, Kim SU, et al. Inhibition of tumour angiogenesis and growth by small hairpin HIF-1alpha and IL-8 in hepatocellular carcinoma. Liver Int 2014;34(4):632–42.Zhao L, Zhang C, Liao G, Long J. RNAi-mediated inhibition of PDGF-D leads to decreased cell growth, invasion and angiogenesis in the SGC-7901 gastric cancer xenograft model. Cancer Biol Ther 2010;9(1):42–8.Appelmann I, Liersch R, Kessler T, Mesters RM, Berdel WE. Angiogenesis inhibition in cancer therapy: platelet-derived growth factor (PDGF) and vascular endothelial growth factor (VEGF) and their receptors: biological functions and role in malignancy. Recent Results Cancer Res 2010;180:51–81.Lazzeri S, Orlandi P, Piaggi P, Sartini MS, Casini G, Guidi G, et al. IL-8 and VEGFR-2 polymorphisms modulate long-term functional response to intravitreal ranibizumab in exudative age-related macular degeneration. Pharmacogenomics 2016;17(1):35–9.Zhang B, Finn RS. Personalized clinical trials in hepatocellular carcinoma based on biomarker selection. Liver Cancer 2016;5(3):221–32.Moriguchi M, Umemura A, Itoh Y. Current status and future prospects of chemotherapy for advanced hepatocellular carcinoma. Clin J Gastroenterol 2016;9(4):184–90.Fulda S, Debatin KM. HIF-1-regulated glucose metabolism: a key to apoptosis resistance? Cell Cycle 2007;6(7):790–2.Tzouvelekis A, Ntolios P, Karameris A, Koutsopoulos A, Boglou P, Koulelidis A, et al. Expression of hypoxia-inducible factor (HIF)-1a-vascular endothelial growth factor (VEGF)-inhibitory growth factor (ING)-4-axis in sarcoidosis patients. BMC Res Notes 2012;5:654.Eckle T, Kewley EM, Brodsky KS, Tak E, Bonney S, Gobel M, et al. Identification of hypoxia-inducible factor HIF-1A as transcriptional regulator of the A2B adenosine receptor during acute lung injury. J Immunol 2014;192(3):1249–56.Kitajima Y, Miyazaki K. The critical impact of HIF-1a on gastric cancer biology. Cancers (Basel) 2013;5(1):15–26.Minet E, Michel G, Remacle J, Michiels C. Role of HIF-1 as a transcription factor involved in embryonic development, cancer progression and apoptosis (review). Int J Mol Med 2000;5(3):253–9.Yu JX, Cui L, Zhang QY, Chen H, Ji P, Wei HJ, et al. Expression of NOS and HIF-1alpha in human colorectal carcinoma and implication in tumor angiogenesis. World J Gastroenterol 2006;12(29):4660–4.Nguyen LK, Cavadas MA, Scholz CC, Fitzpatrick SF, Bruning U, Cummins EP, et al. A dynamic model of the hypoxia-inducible factor 1a (HIF-1a) network. J Cell Sci 2015;128(2):422.Nishimoto A, Kugimiya N, Hosoyama T, Enoki T, Li TS, Hamano K. HIF-1alpha activation under glucose deprivation plays a central role in the acquisition of anti-apoptosis in human colon cancer cells. Int J Oncol 2014;44(6):2077–84.Pan XY, Zhang ZH, Wu LX, Wang ZC. Effect of HIF-1a/VEGF signaling pathway on plasma progesterone and ovarian prostaglandin F(2)a secretion during luteal development of pseudopregnant rats. Genet Mol Res 2015;14(3):8796–809.Jones MK, Szabo IL, Kawanaka H, Husain SS, Tarnawski AS. von Hippel Lindau tumor suppressor and HIF-1alpha: new targets of NSAIDs inhibition of hypoxia-induced angiogenesis. FASEB J 2002;16(2):264–6.Harada A, Sekido N, Akahoshi T, Wada T, Mukaida N, Matsushima K. Essential involvement of interleukin-8 (IL-8) in acute inflammation. J Leukoc Biol 1994;56(5):559–64.Zhang W, Chen H. The study on the interleukin-8 (IL-8). Sheng Wu Yi Xue Gong Cheng Xue Za Zhi 2002;19(4):697–702.David JM, Dominguez C, Hamilton DH, Palena C. The IL-8/IL-8R axis: a double agent in tumor immune resistance. Vaccines (Basel) 2016;4(3).Balkwill F. Cancer and the chemokine network. Nat Rev Cancer 2004;4(7):540–50.Baggiolini M, Walz A, Kunkel SL. Neutrophil-activating peptide-1/interleukin 8, a novel cytokine that activates neutrophils. J Clin Invest 1989;84(4):1045–9.Brinkmann V, Reichard U, Goosmann C, Fauler B, Uhlemann Y, Weiss DS, et al. Neutrophil extracellular traps kill bacteria. Science 2004;303(5663):1532–5.Sakamoto Y, Harada T, Horie S, Iba Y, Taniguchi F, Yoshida S, et al. Tumor necrosis factor-alpha-induced interleukin-8 (IL-8) expression in endometriotic stromal cells, probably through nuclear factor-kappa B activation: gonadotropin-releasing hormone agonist treatment reduced IL-8 expression. J Clin Endocrinol Metab 2003;88(2):730–5.Schraufstatter IU, Trieu K, Zhao M, Rose DM, Terkeltaub RA, Burger M. IL-8-mediated cell migration in endothelial cells depends on cathepsin B activity and transactivation of the epidermal growth factor receptor. J Immunol 2003;171(12):6714–22.Wang L, Tang C, Cao H, Li K, Pang X, Zhong L, et al. Activation of IL-8 via PI3K/Akt-dependent pathway is involved in leptin-mediated epithelial–mesenchymal transition in human breast cancer cells. Cancer Biol Ther 2015;16(8):1220–30.Xie Q, Sun Z, Chen M, Zhong Q, Yang T, Yi J. IL-8 up-regulates proliferative angiogenesis in ischemic myocardium in rabbits through phosphorylation of Akt/GSK-3beta(ser9) dependent pathways. Int J Clin Exp Med 2015;8(8):12498–508.Sasabe E, Tatemoto Y, Li D, Yamamoto T, Osaki T. Mechanism of HIF-1alpha-dependent suppression of hypoxia-induced apoptosis in squamous cell carcinoma cells. Cancer Sci 2005;96(7):394–402.Cao Y. Tumor angiogenesis and therapy. Biomed Pharmacother 2005;59 Suppl 2:S340–3.Chen QR, Zhang L, Gasper W, Mixson AJ. Targeting tumor angiogenesis with gene therapy. Mol Genet Metab 2001;74(1–2):120–7.Varda-Bloom N, Shaish A, Gonen A, Levanon K, Greenbereger S, Ferber S, et al. Tissue-specific gene therapy directed to tumor angiogenesis. Gene Ther 2001;8(11):819–27.Cho SW, Kitisin K, Buck D, Steel J, Brufsky A, Gillespie R, et al. Transcatheter arterial chemoembolization is a feasible palliative locoregional therapy for breast cancer liver metastases. Int J Surg Oncol 2010;2010:251621.Goda N, Dozier SJ, Johnson RS. HIF-1 in cell cycle regulation, apoptosis, and tumor progression. Antioxid Redox Signal 2003;5(4):467–73.Choi SH, Park JY, Kang W, Kim SU, Kim DY, Ahn SH, et al. Knockdown of HIF-1alpha and IL-8 induced apoptosis of hepatocellular carcinoma triggers apoptosis of vascular endothelial cells. Apoptosis 2015. doi:10.1007/s10495-015-1185-2.Choi SH, Chung AR, Kang W, Park JY, Lee MS, Hwang SW, et al. Silencing of hypoxia-inducible factor-1beta induces anti-tumor effects in hepatoma cell lines under tumor hypoxia. PLoS One 2014;9(7):e103304.Barata P, Sood AK, Hong DS. RNA-targeted therapeutics in cancer clinical trials: current status and future directions. Cancer Treat Rev 2016;50:35–47.Aagaard L, Rossi JJ. RNAi therapeutics: principles, prospects and challenges. Adv Drug Deliv Rev 2007;59(2–3):75–86.Singh S, Narang AS, Mahato RI. Subcellular fate and off-target effects of siRNA, shRNA, and miRNA. Pharm Res 2011;28(12):2996–3015.Karpala AJ, Doran TJ, Bean AG. Immune responses to dsRNA: implications for gene silencing technologies. Immunol Cell Biol 2005;83(3):211–6.Rao DD, Vorhies JS, Senzer N, Nemunaitis J. siRNA vs. shRNA: similarities and differences. Adv Drug Deliv Rev 2009;61(9):746–59.Bakhtiyari S, Haghani K, Basati G, Karimfar MH. siRNA therapeutics in the treatment of diseases. Ther Deliv 2013;4(1):45–57.Khatri N, Rathi M, Baradia D, Trehan S, Misra A. In vivo delivery aspects of miRNA, shRNA and siRNA. Crit Rev Ther Drug Carrier Syst 2012;29(6):487–527.Lam JK, Chow MY, Zhang Y, Leung SW. siRNA versus miRNA as therapeutics for gene silencing. Mol Ther Nucleic Acids 2015;4:e252.Lam JK, Liang W, Chan HK. Pulmonary delivery of therapeutic siRNA. Adv Drug Deliv Rev 2012;64(1):1–15.Ozcan G, Ozpolat B, Coleman RL, Sood AK, Lopez-Berestein G. Preclinical and clinical development of siRNA-based therapeutics. Adv Drug Deliv Rev 2015;87:108–19.Fujita Y, Kuwano K, Ochiya T. Development of small RNA delivery systems for lung cancer therapy. Int J Mol Sci 2015;16(3):5254–70.Rolle K, Nowak S, Wyszko E, Nowak M, Zukiel R, Piestrzeniewicz R, et al. Promising human brain tumors therapy with interference RNA intervention (iRNAi). Cancer Biol Ther 2010;9(5):396–406.Li J, Liu J, Li S, Hao Y, Chen L, Zhang X. Antibody h-R3-dendrimer mediated siRNA has excellent endosomal escape and tumor targeted delivery ability, and represents efficient siPLK1 silencing and inhibition of cell proliferation, migration and invasion. Oncotarget 2016;7(12):13782–96.Zhao J, Mi Y, Feng SS. Targeted co-delivery of docetaxel and siPlk1 by herceptin-conjugated vitamin E TPGS based immunomicelles. Biomaterials 2013;34(13):3411–21.Golan T, Khvalevsky EZ, Hubert A, Gabai RM, Hen N, Segal A, et al. RNAi therapy targeting KRAS in combination with chemotherapy for locally advanced pancreatic cancer patients. Oncotarget 2015;6(27):24560–70.Aleku M, Schulz P, Keil O, Santel A, Schaeper U, Dieckhoff B, et al. Atu027, a liposomal small interfering RNA formulation targeting protein kinase N3, inhibits cancer progression. Cancer Res 2008;68(23):9788–98.Matsumoto G, Kushibiki T, Kinoshita Y, Lee U, Omi Y, Kubota E, et al. Cationized gelatin delivery of a plasmid DNA expressing small interference RNA for VEGF inhibits murine squamous cell carcinoma. Cancer Sci 2006;97(4):313–21.Qi L, Xing LN, Wei X, Song SG. Effects of VEGF suppression by small hairpin RNA interference combined with radiotherapy on the growth of cervical cancer. Genet Mol Res 2014;13(3):5094–106.Kanasty R, Dorkin JR, Vegas A, Anderson D. Delivery materials for siRNA therapeutics. Nat Mater 2013;12(11):967–77.Ha M, Kim VN. Regulation of microRNA biogenesis. Nat Rev Mol Cell Biol 2014;15(8):509–24.Treiber T, Treiber N, Meister G. Regulation of microRNA biogenesis and function. Thromb Haemost 2012;107(4):605–10.Shu D, Li H, Shu Y, Xiong G, Carson WE, 3rd, Haque F, et al. Systemic delivery of anti-miRNA for suppression of triple negative breast cancer utilizing RNA nanotechnology. ACS Nano 2015;9(10):9731–40.Liang J, Huang W, Cai W, Wang L, Guo L, Paul C, et al. Inhibition of microRNA-495 enhances therapeutic angiogenesis of human induced pluripotent stem cells. Stem Cells 2016. doi:10.1002/stem.2477.Cao M, Nie W, Li J, Zhang Y, Yan X, Guan X, et al. MicroRNA-495 induces breast cancer cell migration by targeting JAM-A. Protein Cell 2014;5(11):862–72.Ma D, Lu H, Qu Y, Fu W, Ma Z. Developing an effective therapeutic by delivery of synthetic microRNA-520e in lung cancer treatment. Biomed Pharmacother 2015;69:249–54.Semenza GL. The hypoxic tumor microenvironment: a driving force for breast cancer progression. Biochim Biophys Acta 2015. doi:10.1016/j.bbamcr.2015.05.036.Semenza GL. Vasculogenesis, angiogenesis, and arteriogenesis: mechanisms of blood vessel formation and remodeling. J Cell Biochem 2007;102(4):840–7.Zagzag D, Friedlander DR, Margolis B, Grumet M, Semenza GL, Zhong H, et al. Molecular events implicated in brain tumor angiogenesis and invasion. Pediatr Neurosurg 2000;33(1):49–55.Hirota K, Semenza GL. Regulation of angiogenesis by hypoxia-inducible factor 1. Crit Rev Oncol Hematol 2006;59(1):15–26.Semenza GL. Regulation of hypoxia-induced angiogenesis: a chaperone escorts VEGF to the dance. J Clin Invest 2001;108(1):39–40.Plate KH. Gene therapy of malignant glioma via inhibition of tumor angiogenesis. Cancer Metastasis Rev 1996;15(2):237–40.Sato Y. Molecular diagnosis of tumor angiogenesis and anti-angiogenic cancer therapy. Int J Clin Oncol 2003;8(4):200–6.Natarajan R, Fisher BJ, Fowler AA, 3rd. Hypoxia inducible factor-1 modulates hemin-induced IL-8 secretion in microvascular endothelium. Microvasc Res 2007;73(3):163–72.Fernando RI, Castillo MD, Litzinger M, Hamilton DH, Palena C. IL-8 signaling plays a critical role in the epithelial-mesenchymal transition of human carcinoma cells. Cancer Res 2011;71(15):5296–306.Xie TX, Xia Z, Zhang N, Gong W, Huang S. Constitutive NF-kappaB activity regulates the expression of VEGF and IL-8 and tumor angiogenesis of human glioblastoma. Oncol Rep 2010;23(3):725–32.Bohensky J, Shapiro IM, Leshinsky S, Terkhorn SP, Adams CS, Srinivas V. HIF-1 regulation of chondrocyte apoptosis: induction of the autophagic pathway. Autophagy 2007;3(3):207–14.Volm M, Koomagi R. Hypoxia-inducible factor (HIF-1) and its relationship to apoptosis and proliferation in lung cancer. Anticancer Res 2000;20(3A):1527–33.Xu K, Ding Q, Fang Z, Zheng J, Gao P, Lu Y, et al. Silencing of HIF-1alpha suppresses tumorigenicity of renal cell carcinoma through induction of apoptosis. Cancer Gene Ther 2010;17(3):212–22.Jawahir M, Nicholas SA, Coughlan K, Sumbayev VV. Apoptosis signal-regulating kinase 1 (ASK1) and HIF-1alpha protein are essential factors for nitric oxide-dependent accumulation of p53 in THP-1 human myeloid macrophages. Apoptosis 2008;13(12):1410–6.Greijer AE, van der Wall E. The role of hypoxia inducible factor 1 (HIF-1) in hypoxia induced apoptosis. J Clin Pathol 2004;57(10):1009–14.Sendoel A, Kohler I, Fellmann C, Lowe SW, Hengartner MO. HIF-1 antagonizes p53-mediated apoptosis through a secreted neuronal tyrosinase. Nature 2010;465(7298):577–83.Xia L, Mo P, Huang W, Zhang L, Wang Y, Zhu H, et al. The TNF-alpha/ROS/HIF-1-induced upregulation of FoxMI expression promotes HCC proliferation and resistance to apoptosis. Carcinogenesis 2012;33(11):2250–9.Khan MN, Bhattacharyya T, Andrikopoulos P, Esteban MA, Barod R, Connor T, et al. Factor inhibiting HIF (FIH-1) promotes renal cancer cell survival by protecting cells from HIF-1alpha-mediated apoptosis. Br J Cancer 2011;104(7):1151–9.Horree N, Groot AJ, van Hattem WA, Heintz AP, Vooijs M, van Diest PJ. HIF-1A gene mutations associated with higher microvessel density in endometrial carcinomas. Histopathology 2008;52(5):637–9.Cursio R, Miele C, Filippa N, Van Obberghen E, Gugenheim J. Liver HIF-1 alpha induction precedes apoptosis following normothermic ischemia–reperfusion in rats. Transplant Proc 2008;40(6):2042–5.Gupta-Saraf P, Miller CL. HIF-1alpha downregulation and apoptosis in hypoxic prostate tumor cells infected with oncolytic mammalian orthoreovirus. Oncotarget 2014;5(2):561–74.Ueda M, Ueki K, Kumagai K, Terai Y, Okamoto Y, Ueki M, et al. Apoptosis and tumor angiogenesis in cervical cancer after preoperative chemotherapy. Cancer Res 1998;58(11):2343–6.Albertsson P, Lennernas B, Norrby K. On metronomic chemotherapy: modulation of angiogenesis mediated by VEGE-A. Acta Oncol 2006;45(2):144–55.Gasparini G, Biganzoli E, Bonoldi E, Morabito A, Fanelli M, Boracchi P. Angiogenesis sustains tumor dormancy in patients with breast cancer treated with adjuvant chemotherapy. Breast Cancer Res Treat 2001;65(1):71–5.Jijon HB, Buret A, Hirota CL, Hollenberg MD, Beck PL. The EGF receptor and HER2 participate in TNF-alpha-dependent MAPK activation and IL-8 secretion in intestinal epithelial cells. Mediators Inflamm 2012;2012:207398.Petzelbauer P, Watson CA, Pfau SE, Pober JS. IL-8 and angiogenesis: evidence that human endothelial cells lack receptors and do not respond to IL-8 in vitro. Cytokine 1995;7(3):267–72.Chen C, Yu Z. siRNA targeting HIF-1alpha induces apoptosis of pancreatic cancer cells through NF-kappaB-independent and -dependent pathways under hypoxic conditions. Anticancer Res 2009;29(4):1367–72.Shive HR, West RR, Embree LJ, Sexton JM, Hickstein DD. Expression of KRASG12V in zebrafish gills induces hyperplasia and CXCL8-associated inflammation. Zebrafish 2015;12(3):221–9.Grabner B, Schramek D, Mueller KM, Moll HP, Svinka J, Hoffmann T, et al. Disruption of STAT3 signalling promotes KRAS-induced lung tumorigenesis. Nat Commun 2015;6:6285.


## References

[1] Choi SH, Park JY (2017). Regulation of the hypoxic tumor environment in hepatocellular carcinoma using RNA interference. Cancer Cell Int.

